# Allopurinol use in pregnancy in three women with inflammatory bowel disease: safety and outcomes: a case series

**DOI:** 10.1186/1471-230X-13-172

**Published:** 2013-12-17

**Authors:** Muhammad W Fazal, Matt P Doogue, Rupert W Leong, Peter A Bampton, Jane M Andrews

**Affiliations:** 1 Internal Medicine Service, Royal Adelaide Hospital, Adelaide, SA 5000, Australia; 2 Endocrinology/Clinical Pharmacology, Flinders University School of Medicine and Flinders Medical Centre, Bedford Park, SA 5042, Australia; 3 Gastroenterology and Liver Services, Sydney Local Health District, Concord Hospital, Sydney, NSW 2139, Australia; 4 Department of Gastroenterology and Hepatology, Flinders Medical Centre, Bedford Park, SA 5042, Australia; 5 IBD Service, Department of Gastroenterology & Hepatology and School of Medicine at Royal Adelaide Hospital, Adelaide, SA 5000, Australia

**Keywords:** Allopurinol, Pregnancy, Teratogen, Inflammatory bowel disease, Ulcerative colitis, Azathioprine, 6-mercaptopurine

## Abstract

**Background:**

Allopurinol is a frequently prescribed drug. In inflammatory bowel disease patients who shunt thiopurine metabolism towards more toxic and less desirable pathways, allopurinol is proving to be an effective add on therapy with good resultant disease control and less treatment side effects. As many such patients are young, the potential for pregnant women to be exposed to allopurinol is increasing. The safety of allopurinol in pregnancy is not known however.

**Case presentation:**

We report three cases of safe use of allopurinol in pregnancy for women with inflammatory bowel disease. This included 2 patients with ulcerative colitis and 1 patient with fistulising Crohn’s disease. Allopurinol was used throughout pregnancy in all patients. All 3 pregnancies resulted in normal healthy babies born at term by Caesarean section.

**Conclusion:**

It is important to evaluate and document the safety of allopurinol during pregnancy, as it is finding new roles in young patients. These three cases add significantly to the very limited data on allopurinol use in pregnancy. We encourage reporting of all cases of allopurinol use in pregnant patients and suggest an allopurinol pregnancy registry to document drug exposures and outcomes.

## Background

Allopurinol was discovered by Gertrude Elion in the late 1950’s in studies intended to improve thiopurine efficacy [1]. Since registration by the FDA in 1966 it has been predominantly used to treat patients with hyperuricaemia. Most such patients are either male, beyond child-bearing years or undergoing chemotherapy, and hence potential teratogenicity of allopurinol has not been a concern. However, allopurinol is increasingly prescribed to younger female patients and there is minimal information on allopurinol safety in pregnancy.

There are several reasons for the increasing prescription of allopurinol to younger female patients. With increasing obesity gout is increasingly seen in women of child-bearing age. Leukaemia is a common malignancy in the young and chemotherapy induced hyperuricemia is treated with allopurinol. Furthermore, allopurinol use is expanding beyond hyperuricaemia to combination use with thiopurines (6-mercaptopurine or azathioprine) in some cases of inflammatory bowel disease (IBD) and autoimmune hepatitis.. Allopurinol is used in this situation to promote the metabolism of azathioprine/6-mercaptopurine along therapeutically favourable pathways in cases where preferential production of 6 methyl mercaptopurine (6MMP) rather than 6 thioguanine nucleotides (6TGN) has lead to treatment failure or toxicity [2-4]. Since these patients are frequently young and female, this indication has also expanded the use of allopurinol in potentially fertile women.

Allopurinol is categorised pregnancy risk “C” by the FDA as it inhibits purine synthesis and hence may have direct affects on dividing cells in utero. It is excreted in breast milk and caution is advised when used in nursing mothers. Animal studies with allopurinol have shown variable results. One study in 1972 reported cleft palate and skeletal abnormalities in mice embryos exposed to large doses of allopurinol [5]. However, studies in other animal species report no foetal harm [6]. Published human data are scarce but growing. Just recently Hoeltzenbein et al. have reported case series of 31 prospectively determined pregnancies with 1^st^ trimester allopurinol exposure. The rate of major malformations was in the expected range, however one infant had severe malformations including microphthalmia, cleft lip and palate, renal hypoplasia, low-set ears, hearing deficit, bilateral cryptorchidism, and micropenis [7]. This phenotype is of possible concern as it resembles another reported case of multiple congenital anomalies in an allopurinol exposed infant [8]. These reports have raised concerns regarding potential teratogenicity of allopurinol. However safe use of allopurinol and mercaptopurine in a pregnant ulcerative colitis patient has been reported with a resultant favourable pregnancy outcome [9]. There are also four cases reported of allopurinol use in pregnant women with leukaemia. Two of these pregnancies resulted in normal healthy infants and two in adverse outcomes (intrauterine growth retardation and intrauterine foetal death), both of which were attributable to other causes [10-12]. In these cases allopurinol was used in the 2^nd^ or 3^rd^ trimester only.

Although it is likely that there have been exposures to allopurinol in multiple pregnancies, very few have been reported as mentioned above. We report the safe use of allopurinol through three pregnancies in mothers on thiopurines for IBD.

## Case presentation

### Case 1

A 29 year old woman diagnosed with ulcerative colitis in July 2007 had several hospital admissions over the following 18 months requiring IV steroids and cyclosporine for acute ulcerative colitis despite good reported compliance with mesalazine and azathioprine. In 2009 thiopurine metabolites were assessed (newly available) and she was documented to have a low 6TGN and a 6MMP:6TGN ratio of 51 consistent with MMP preferential shunting. She was commenced on allopurinol 100 mg PO daily and the dose of azathioprine reduced to 50 mg PO daily. Subsequently she was weaned off steroids and cyclosporine, remaining clinically stable on allopurinol and azathioprine. One year after addition of allopurinol in 2010, her 6MMP:6TGN ratio was 0.25.

In 2011 she conceived her first child and at about 10 weeks gestation had a flare of ulcerative colitis requiring admission. On arrival, it was apparent that she had a colonic perforation secondary to severe ulcerative colitis. An urgent subtotal colectomy with end ileostomy was performed. First trimester ultrasound revealed a healthy foetus at expected gestational age. She was maintained on allopurinol 100 mg and azathioprine 75 mg PO daily postoperatively due to concerns about disease control in the long rectal stump. At the time it was considered that the majority of the risk of teratogenic effects of allopurinol had already occurred, and that the potential benefit in controlling disease for the remainder of the pregnancy outweighed the potential harm of ongoing drug exposure. She was discharged 20 days later after good clinical recovery.

Her pregnancy progressed unremarkably. She underwent an emergency Caesarean section at 37 weeks gestation following spontaneous rupture of membranes. She delivered a healthy 3 kg baby boy. He had an inguinal hernia repair at 5 weeks and remains well since then.

### Case 2

A 29 year old woman was referred with steroid dependent ulcerative colitis diagnosed 2 years earlier. She was treated with prednisolone 40 mg PO daily, azathioprine 150 mg PO daily, mesalazine tablets 2 g PO twice daily and mesalazine foam enemas 4 g PR daily. She was also taking folate 0.5 mg PO daily as she was attempting to conceive. Her TPMT (thiopurine methyltransferase) activity before therapy was normal at 8.5U/mL (7.0 – 14.5). In evaluating her steroid dependency her 6TGN was found to be 134 with 6MMP 5560, giving a 6MMP:6TGN ratio of 41, consistent with shunting. It was judged that an increased azathioprine dose would have lead to a large 6MMP rise (with such a ratio) and probable toxicity, so co-therapy was decided on. The azathioprine dose was reduced to 25 mg PO daily and allopurinol 100 mg PO daily was dosed concomitantly. She declined treatment with a biological agent or entry into a clinical drug trial as she was attempting to conceive.

She conceived 15 months later on the allopurinol & azathioprine co-therapy. The prednisolone dose was reduced to 7.5 mg PO daily; the lowest dose she had been on. To avoid a flare during pregnancy the decision was to maintain her at low dose prednisolone. At the time, her 6TGN was 254 and 6MMP was < 500 (6MMP:6TGN ratio ~2). Allopurinol, azathioprine and prednisolone were continued for the duration of the pregnancy and her ulcerative colitis was well controlled.

A healthy baby boy was delivered by caesarean section at term due to breech position. She elected not to breast feed for personal reasons. Sixteen months after delivery of her first child she suffered a miscarriage at 6.5 weeks pregnancy. Foetal chromosomal studies were not performed. She plans to fall pregnant again in the following year before entering a clinical trial to manage her ulcerative colitis. She continues on combination azathioprine and allopurinol.

### Case 3

A 28 year old woman presented in year 2000 with an ischiorectal abscess at age 17. This was drained after which she developed a fistula, which was managed with a cable tie seton before resolving. She re-presented in 2002 with obstructive symptoms and a small bowel series revealed stenosing ileocaecal Crohn’s disease. From 2002 and 2008 she was managed with azathioprine 150 mg PO daily with intermittent steroid courses and one induction dose of infliximab (prior to funded access to therapy).

In June 2008, she was commenced on induction and maintenance therapy with adalimumab for recurrent perineal fistulae, remaining on concomitant azathioprine 150 mg PO daily. In Feb 2009, she ceased azathioprine as she was in clinical remission. However in June 2009, a flare requiring a course of prednisolone occurred and azathioprine 150 mg PO daily was recommenced. Prednisolone was ceased, but she remained mildly symptomatic. Metabolite testing at this time revealed a 6TGN of 181 and a 6MMP of 4558 (6MMP:6TGN ratio of 25). Her dose was gradually increased to azathioprine 225 mg PO daily, however, repeat testing revealed 6 TGN 253 and 6 MMP 8093; a ratio of 32, consistent with shunting. Whilst liver function tests, iron and inflammatory markers were all normal, the patient complained of lethargy and nausea. She was placed on concomitant allopurinol 100 mg PO daily and the azathioprine dose was reduced to 50 mg PO daily, together with continued adalimumab. A repeat metabolite testing revealed 6TGN of 493 and 6MMP of < 250 with resolution of nausea and lethargy. In March 2011 she reported feeling the best she had ever felt. She subsequently conceived in October 2011. She decided to cease adalimumab at that stage, but remained on azathioprine and allopurinol throughout her pregnancy with no further flares. She delivered a healthy baby boy by elective caesarean section at 38 weeks, and has remained in clinical remission on last review in September 2012.

## Conclusion

Despite 50 years of clinical use there are very scant data to date regarding the use of allopurinol in pregnancy. Notably allopurinol has found new applications particularly in combination with thiopurines. The use of allopurinol in conjunction with thiopurines was reported in cadaveric renal transplants with favourable outcome in terms of reduced rejection episodes [13]. It is now also being used in this fashion in an increasing number of IBD patients i.e. those with preferential production of 6MMP over 6TGN [2,3]. These patients are referred to as “shunters” [4] and co-treatment with allopurinol (pioneered recently in the management of IBD) decreases 6-MMP and increases 6-TGN concentrations to achieve “therapeutic” concentrations of 6TGN; hence a greater chance of effective disease control [14,15]. When used as co-therapy in this setting, allopurinol is continued long term to maintain a desirable profile of 6MMP:6TGN. These patients are often young and female; consequently allopurinol is now being prescribed to a greater number of potentially fertile IBD women as a long-term therapy. The inhibition of purine metabolism by allopurinol raises concerns of teratogenicity, but as yet there appear to be only 2 cases with adverse pregnancy outcomes possibly related to allopurinol [7,8] albeit with uncertain causality.

Women with IBD have an increased risk of adverse pregnancy outcomes (odds ratio, 1.65; 95% confidence interval, 1.09-2.48) [16,17]. The main adverse outcomes are of impaired foetal growth and difficulties with maintenance of pregnancy, with a greater incidence of premature births and low birth weight infants reported in women with IBD [16]. However, the activity of IBD during pregnancy appears to be the most important determinant of these adverse outcomes rather than drug therapy to control IBD; moreover activity of IBD during pregnancy is predominantly determined by the activity of disease at conception [18]. Hence most authors emphasize the importance of optimal disease control pre-conception, and continuing necessary medication during pregnancy.

Therapeutic options can be limited for IBD patients, as many do not maintain remission on a 5-aminosalicylates, and long-term steroid therapy is not acceptable due to cumulative toxicity and frequent lack of mucosal healing. Anti-TNF therapy (anti-tumour necrosis factor) does not always succeed and for many patients there are contraindications to these agents. In addition, in some jurisdictions anti-TNF drugs are not widely available due to funding constraints. In ulcerative colitis surgery is “curative”, yet it is not desired by most young patients, and surgery has its own morbidity and longer term effects. Thiopurines are pregnancy category D and their use has been associated with preterm birth (perhaps as a marker of worse disease) but not with low birth weight or congenital abnormalities [19], and they are therefore widely regarded as the treatment of choice in pregnant patients with IBD that is difficult to control with 5-aminosalicylates [20,21]. Whilst Jharap B et al. have recently reported some concerns in infants born to IBD mothers on thiopurines [22], these data do not appear to have translated into a significant clinical problem for IBD mothers and babies, and allopurinol therapy should not add to the observed anaemias in infants unless higher than recommended 6TGNs are allowed.

With the rise in IBD and the increasing use of concomitant allopurinol with thiopurine treatment, the use of allopurinol in women of child-bearing potential is growing. For example of ~800 IBD patients at the Royal Adelaide Hospital, ~50% are on thiopurines and 15% of thiopurine users have been identified as “shunters”. Nearly 50% of these shunters are women younger than 50. Given the understandable reticence of doctors and potential parents to expose infants to possibly dangerous drugs, good clinical and experimental data to better quantify the teratogenicity of allopurinol is essential. These three cases add significantly to the very limited data on allopurinol use in pregnancy in general and in pregnant IBD patients on thiopurines in particular. The largest case series to date examining allopurinol safety in pregnancy by Hoeltzenbein et al. cited earlier, provides both reassurance (in that the absolute rate of congenital abnormalities was only as expected) and concern (with the affected infant having a constellation of abnormalities similar to another reported allopurinol-exposed case). One should be aware however that none of the cases in the German series were patients with IBD, and all of the women were multiply medicated and with major comorbidities. Moreover, there is likely to be a bias as they all resulted from referrals to a clinical teratology and drug risk assessment clinic. As our cases only came to light post delivery in the 1^st^ case, when one author (JMA) questioned colleagues, thus leading to the discovery of the other cases, we believe it is likely that many other cases with good outcomes are currently un-reported. Thus, until a greater number of exposed pregnancies are reported, it is uncertain what the real rate and scope of any danger is.

In response to a question at Digestive Diseases Week 2012 during a session on drugs in pregnancy, invited speakers and a large audience were unable to make any particular comment on allopurinol experience and outcomes in this setting (JM Andrews personal communication). For many newer drugs there are registries collecting data on drug exposure during pregnancy and outcomes. However, there is a lack of similar initiatives for older drugs with sparse safety data, like allopurinol. Therefore we encourage the reporting of all cases of allopurinol use in pregnancy and the resultant outcomes in the exposed infants. No pregnancy is risk free – even without a chronic disease or drug therapy; however pregnancy is an emotional charged issue. Therefore we would recommend that pre-pregnancy counselling of all IBD patients takes place, addressing both risks of therapy and of active disease, and that patients and their treating team make decisions in a collaborative fashion with the best currently available information. As treatments evolve, prospective follow-up of offspring from IBD-pregnancies should be resourced.

### Consent

Written informed consents were obtained from the patients for publication of this case series. A copy of the written consent is available for review by the editor of this journal.

## Abbreviations

IBD: Inflammatory bowel disease; 6MMP: 6 methyl mercaptopurine; 6TGN: 6 thioguanine nucleotide; Anti-TNF: Anti-tumor necrosis factor; TPMT: Thiopurine methyltransferase.

## Competing interests

The authors declare that they have no competing interests.

## Authors’ contributions

MWF contributed to the data search, drafting and critical revision of the transcript. MD contributed significantly to data search and analysis and interpretation of data. RL made contribution to acquisition of data. PB helped with drafting, acquisition of data and revision. JMA contributed significantly to conception and design, acquisition of data, drafting and critical revision of transcript. All authors read and approved the final version of the transcript.

## Pre-publication history

The pre-publication history for this paper can be accessed here:

http://www.biomedcentral.com/1471-230X/13/172/prepub
